# Genetic species identification of ecologically important planthoppers (*Prokelisia* spp.) of coastal *Spartina* saltmarshes using High Resolution Melting Analysis (HRMA)

**DOI:** 10.1038/s41598-019-56518-4

**Published:** 2019-12-27

**Authors:** G. Janelle Espinoza, Jaime R. Alvarado Bremer

**Affiliations:** 1 Texas A&M University at Galveston, Department of Marine Biology, 1001 Texas Clipper Road, Galveston, TX 77554-2888 USA; 2 Texas A&M University, Department of Wildlife and Fisheries Sciences, 210 Nagle Hall, Texas A&M University, College Station, TX 77843-2258 USA

**Keywords:** Genotype, DNA sequencing

## Abstract

Phloem-feeding planthoppers of the genus *Prokelisia* rank among the most abundant and ecologically important browsers of coastal saltmarsh grasses of eastern North America and the Caribbean. Along the *Spartina* marshes of the northern Gulf of Mexico, the sympatric species *P*. *marginata* and *P*. *dolus* are the most abundant, but are difficult to distinguish from each other based solely on morphology. This study seeks to design a molecular assay based on High Resolution Melting Analysis (HRMA) as a fast, cost-effective alternative to differentiate these species. A 450 base pairs (bp) segment of cytochrome c oxidase subunit I (COI) was amplified and sequenced for representative samples of both species, and a short amplicon (SA) HRMA was designed based on the presence of fixed nucleotide differences between species found along a 60 bp segment of COI. The unambiguous identification of individual specimens of *P*. *marginata* or *P*. *dolus* was possible due to easily discernable differences in the melting temperatures of the two species along this mini barcode. This assay may prove useful for future genetic studies involving these species by preventing the overestimation of genetic diversity via inclusion of conspecifics, and in ecological studies by improving data on the effects of individual species of *Prokelisia*.

## Introduction

Members of *Prokelisia* are wing dimorphic, phloem-feeding planthoppers that, along with other species of leafhoppers and mirid bugs, may account for greater than 90% of herbivore biomass in salt marshes^1–3^. Three of the five species of *Prokelisia*, namely *P*. *marginata*, *P*. *dolus*, and *P*. *crocea*, have overlapping geographic ranges along the east coast of North America, from the Gulf of Mexico to Canada, where they have a close association with *Spartina* salt marshes^4^. They rank as the most important browsers, with the capacity to influence plant cover and, consequently, ecosystem services provided by *Spartina* grasses, which include such things as provision of complex habitat, primary production, and carbon sequestration^5–7^. *P*. *crocea*, a species with ampler habitat preference than the other two, is easily distinguished by its larger size and unique orange markings on its face and thorax. By contrast, *P*. *marginata* and *P*. *dolus* are very similar in size and pigmentation patterns, and species identification is based on the frons shape, which in some individuals displays an intermediate condition^8,9^. A more detailed microscopic examination of the male genitalia of these species reveals differences in the shapes of the styles, aedeagus, and anal tube^4^. Preliminary results from this study revealed that identification using this method is time consuming, and the character states of these traits can be easily misdiagnosed. Further, female sex organs of the two species differ from each other, but since they often display intermediate morphological features, species identification of females is unreliable^8^. Also, while juveniles of both species can be assigned to the *Prokelisia* genus, their undeveloped sex organs make it impossible to distinguish their gender or identify them to species level.

Correct species identification is particularly relevant when conducting population studies, and when cryptic or hard-to-distinguish species occur in sympatry^10^. Estimates of allele frequency and the corresponding diversity indices can be severely biased through the inclusion of misidentified specimens^11^. The use of molecular methodologies can facilitate the identification of cryptic species, and new technologies like high resolution melting analysis provide a fast, high-throughput alternative that is less expensive (e.g. ~$0.10 per reaction for the SA-HRMA performed in this study) than other genotyping methodologies^12^. HRMA is capable of detecting single nucleotide polymorphisms (SNPs) and small deletions present within amplified DNA fragments that are visualized as differences in the fluorescence of a saturating dye that is disassociated as the amplicons denature with increasing temperature^13^. A major advantage of HRMA over other genotyping methods is that the entire process, from amplification to scoring, can be completed in approximately 15–20 minutes using modern RT-PCR equipment. Since this occurs in a single, closed-tube assay, the potential for cross contamination is minimized, and there is no need for multiple steps using different platforms to score alleles^12^. HRMA has been successfully used to characterize wild populations^12^, including the identification of marine fishes^12,14^, as well as arthropods and spiders^15^. This study seeks to use HRMA as a novel approach to rapidly distinguish between *P*. *marginata* and *P*. *dolus*.

## Results and Discussion

**Figure 1:**
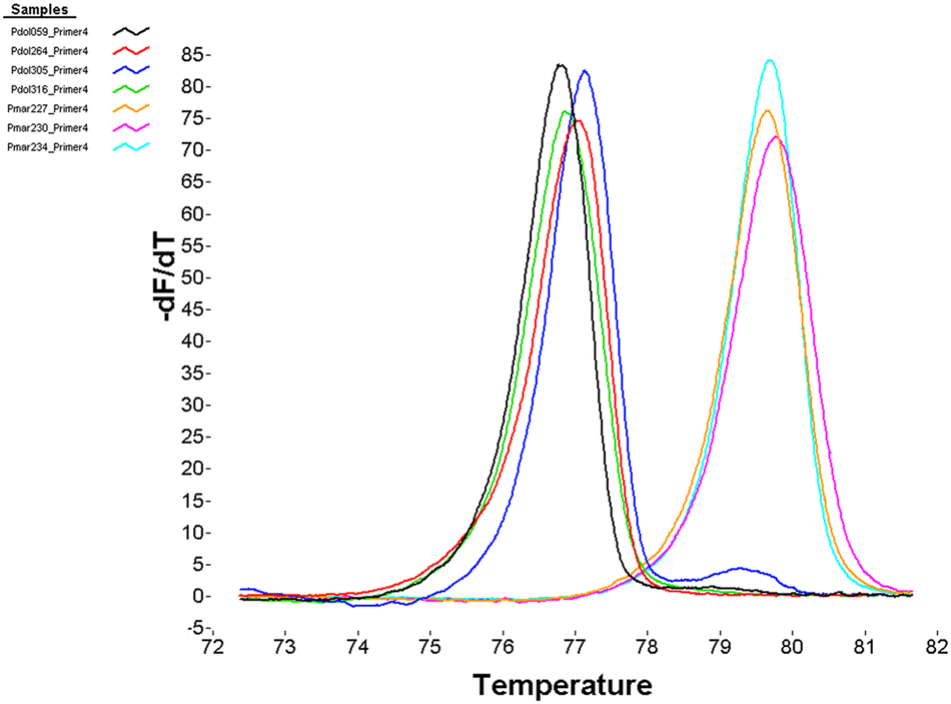
Preliminary results of SA-HRMA using Prok-HRMA4 primer pair. The graph shows normalized derivative plots of fluorescence with respect to temperature (−dF/dT) for a total of seven specimens of *P*. *marginata* (n = 3) and *P*. *dolus* (n = 4) each identified with a different line color. The specimen ID and the primer set employed is given in the inset. *P*. *marginata* haplotypes melted at a higher T_m_ than *P*. *dolus*. Reactions were carried out in a RapidCycler2 and scored in an HR-1 instrument.

**Figure 2:**
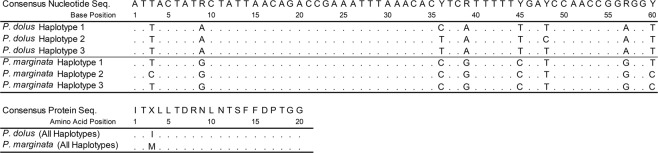
Multiple sequence alignment of the fragment amplified by Prok-HRMA4 primer pair. Alignment includes six planthopper haplotypes belonging to *P*. *marginata* and *P*. *dolus* defined by eight polymorphic sites, and translated amino acid sequences (20 amino acids). The corresponding consensus nucleotide and amino acid sequences are shown. All substitutions were transitions at the third codon position, and all but one (A/G at position 9) were synonymous. The haplotypes shown here correspond to individuals shown in Fig. 1.

Preliminary HRMAs using the HR-1 High Resolution Melter and the primer pair Prok-HRMA4-F and Prok-HRMA4-R to characterize a small sample of validated males of *P*. *marginata* and *P*. *dolus* resulted in a separation (up to 3–4 °C) between the melting peaks of the two species, with *P marginata* melting at a higher temperature (Fig. 1). The Prok-HRMA4 primer-pair amplifies a short (60 bp) COI fragment, whose length is particularly well suited to diagnose SNPs using SA-HRMA, as previous studies have found that amplicons <100 bp result in the highest resolution for genotyping using HRMA^16^. The multiple sequence alignment of this short COI fragment consists of three representative haplotypes per species out of the 65 morphologically validated individuals sequenced, and reveals the presence of eight polymorphic sites. All polymorphic sites correspond to transitions at the third codon position (Fig. 2), of which seven are synonymous. The exception is the A/G transition at the ninth nucleotide position that results in a change of Isoleucine in *P*. *dolus* for Methionine in *P*. *marginata*, both of which are hydrophobic amino acids. Four of the polymorphic sites fail to separate the two species, with the first, third, fifth and eight positions identified as plesiomorphies, as haplotypes of the two species share character states at any of these nucleotide sites. Although these plesiomorphies produced variability in the melting profiles within two species (Fig. 1), they did not affect the diagnostic power of this HRMA given that, on average, the haplotypes of the two species differ by 5.6 substitutions, with four of these fixed and responsible for the minimum (>1.7 °C) observed difference separating the melting peaks of these two species. The higher melting temperature of *P*. *marginata* (~79.4 °C) compared to *P*. *dolus* (~77.0 °C) is due to the presence in *P*. *marginata* of a G or a C in the majority of the polymorphic sites, compared to an A or a T at those sites in *P*. *dolus* (Fig. 2).

**Figure 3:**
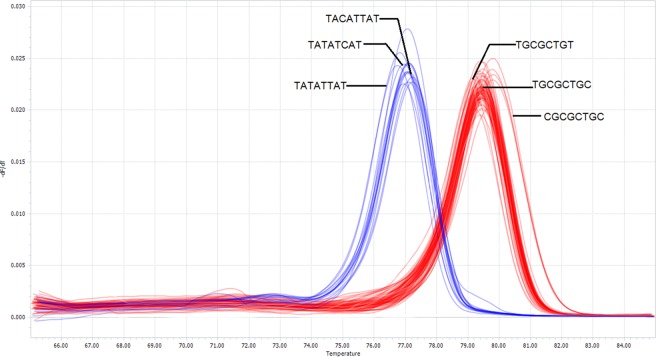
High-throughput SA-HRMA using Prok-HRMA4 primer pair. The graph shows normalized derivative plots of fluorescence with respect to temperature (−dF/dT) for a full plate (n = 96) specimens. Nucleotide sequences correspond to the eight polymorphic sites for each haplotype (See Fig. 1). *P*. *marginata* haplotypes melted at a higher T_m_ than *P*. *dolus*. Reactions were carried out and scored with a LightCycler 96 RT-PCR instrument.

**Figure 4:**
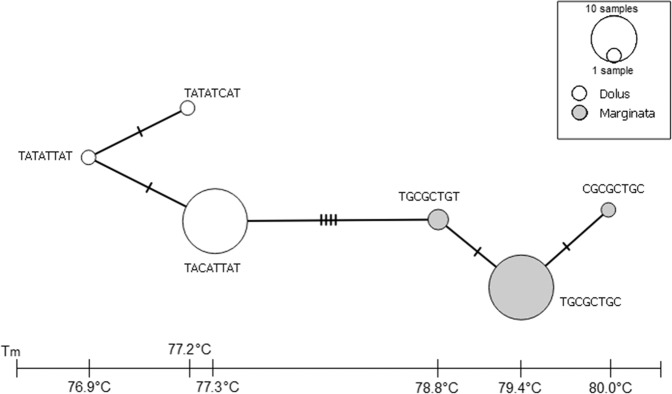
Minimum spanning network for the Prok-HRMA4 primer region for the sequences of the 65 morphologically validated specimens used to develop the HRM assay. Circles correspond to haplotypes, with circle size indicative of the number of individuals belonging to each haplotype. Nucleotide sequences correspond to the eight polymorphic sites for each haplotype. Bottom bar corresponds to the temperatures at which each haplotype melts.

A high throughput analysis (n = 518) using the Prok-HRMA4 primer pair was highly successful with only 1.7% (n = 9) reported amplification failures (Table 1). The successful amplifications (n = 509) produced clearly diagnostic melting curves for the unequivocal assignment of 213 individuals to *P*. *dolus* and 296 individuals to *P*. *marginata* (Fig. 3) in spite of the latter displaying more variation in melting temperatures. Variations in melting temperatures can be attributed to differences in the eight variable sites that make up the six haplotypes, with higher temperatures corresponding to higher presence of G or C at those sites (Fig. 4). A subset of *P*. *marginata* (n = 124) were sequenced for a separate study of population structure in this species. The sequences of the segment targeted by the Prok-HRMA-4 primer set for all those individuals conformed to one of the three haplotypes for *P*. *marginata* shown in this study.

**Table 1:** High-throughput HRMA results for the Prok-HRMA4 primer pair.

	Total	Males	Females
High-Throughput Samples	518	279	239
Failed Amps (%)	9 (1.7%)	9 (3.2%)	0
Ambiguous Curves (%)	0	0	0
Visually Mis-Identified (%)	N/A	30 (10.8%)	N/A

Among the successful samples tested, 279 specimens, roughly half the sample, were males that had been pre-identified to species level based on morphology. Surprisingly, HRMA suggests that 30 individuals, or 10.75% of the male sample (15 *P*. *dolus* and 15 *P*. *marginata*), were misidentified based on putatively diagnostic morphological characters^8,9^. An alternative explanation for the disagreement between morphological and molecular identifications is mitochondrial introgression between *P*. *marginata* and *P*. *dolus*, a phenomenon that has not been reported to date in these insects. Demonstrating this alternative hypothesis would require an extensive analysis of the nuclear genomes and breeding experiments beyond the scope of this study.

The ability to rapidly and efficiently determine the species identity of planthoppers using HRMA, as described here, has important implications to population genetic studies on these species. If such a study were to be conducted relying solely on morphological identification of species, the resultant allele frequency counts would likely be biased due to the inclusion of a high percentage of heterospecifics that would also result in grossly overestimating the genetic diversity of the samples surveyed. Additionally, since the females of these two species are physically intermediate to each other^8^ and juveniles do not display diagnostic characters, any population level study of either species would be limited to the characterization of adult male specimens, hindering the ability to distinguish gender effects from overall population effects. Also, while both species appear to occupy the same niche, and therefore might be grouped in ecological studies under the Ecological Species Concept^17^, that concept requires life histories of the grouped species to be the same, which is not always true in practice^18^. *P*. *dolus* and *P*. *marginata*, in particular, are capable of displaying different life histories even within their individual species, as evidenced by the existence of two wing morphs with different levels of fecundity and dispersal potential that each present under specific environmental conditions and stressors^2,19^. Therefore, ecological studies including these species would also benefit from reliable species identification such that single species effects are not obfuscated by unintentional inclusion of multiple species.

Because of the very small size (2–3 mm) of the *Prokelisia* specimens, entire specimens were digested to provide sufficient DNA quantity (>50 ng/µL) and quality (≥10,000 bp length) to perform this SA-HRMA and subsequent sequencing experiments, including Sanger and next-generation sequencing (NGS). However, it should be noted that arthropods often contain gut endosymbionts that can contaminate massive parallel sequencing experiments^20^. Therefore, investigators wishing to use NGS or RNA-seq techniques on these species should consider isolating nucleic acids from body parts, such as the legs or the head. Finally, it was determined that species identification using this SA-HRMA assay could be successfully carried on different RT-PCR platforms, including Idaho Technology’s RapidCycler-2 and HR-1 machine, Roche’s LightCycler 460 and LightCycler 96, and in a Phoenix’s MyGo Mini, all using the same chemistry and identical, or very similar thermocycling and melting profiles.

## Materials and Methods

### Field collections and sample preparation

Arthropods, including *Prokelisia spp*. specimens, were captured from six *Spartina* marshes along the northern coast of the Gulf of Mexico using professional insect 15″ muslin sweep nets (www.gemplers.com). Immediately after collection, samples were preserved in 70% ethanol (EtOH) and kept there until assayed in the lab. Preserved samples were examined under a dissecting microscope at 10x magnification, and those specimens with the characteristics of both *P*. *dolus* and P. *marginata* were separated from other arthropods. These specimens were further sorted by gender. The number of *Prokelisia* specimens collected per marsh site ranged from 6–200 individuals. In total, 540 individuals, including males, females, and immature of *P*. *dolus* and *P*. *marginata* were collected. Males of the two species were further sorted to species level by examining the putative diagnostic features of their reproductive organs^4^ under a compound microscope at 40x magnification. An example of the different styles shapes seen in *P*. *dolus* and *P*. *marginata* males viewed under a Nikon AZ100M microscope with motorized body and compiled via Nikon BR software is shown in Supplementary Fig. S1.

### HRMA development

A sample consisting of 65 males were identified based on morphological characters, as described above, to belong to *P*. *marginata* (n = 43) or *P*. *dolus* (n = 22). To isolate the DNA, each specimen was individually ground using a sterile disposable pestle, followed by Proteinase K digestion without organic extractions as described by Greig^21^. A 450 bp fragment of the mitochondrial DNA (mtDNA) COI gene was amplified using primers C1-J-1751 and C1-N-2197^22^. COI was targeted because this segment has a proven record to distinguish con-generics of a variety of animal species, including arthropods^23–25^. PCR reactions were carried out in 12.5 µL volumes, containing 1x Econotaq Plus Green Master Mix (Lucigen), 0.2 µM of each primer, and approximately 10 ng of isolated DNA as template. Thermocycling was performed on an Eppendorf Mastercycler (Eppendorf, Hamburg, Germany) with an initial denaturing step at 94 °C for 2 min; followed by 35 cycles of denaturing at 94 °C for 25 sec, annealing at 47 °C for 40 sec, and extension at 72 °C for 90 sec; and a final extension step at 72 °C for 3 minutes. PCR products were then visualized for specificity and yield via electrophoresis on a 2% agarose gel pre-stained with ethidium bromide (EtBr). PCR products that produced a single band were diluted 1:10 for post-PCR cleanup and sequenced in both directions, with reaction setups and thermocycling profiles as described in Cruscanti *et al*.^26^. Multiple sequence alignments were carried out in Geneious Pro v.9.1.8 (Biomatters Ltd., Aukland, NZ).

### Design and evaluation of HRMA

The multiple sequence alignment of 450 bp of COI obtained from the 65 morphologically validated male specimens was used to design a SA-HRMA. Fixed nucleotide differences between species along this segment were identified, and four sets of potential primers, each flanking a short segment (40–106 bp long) containing the diagnostic sites were chosen (Supplementary Fig. S2) following the recommendations given by Smith *et al*.^27^. Preliminary analysis of specificity for each primer set was conducted via PCR amplification using the DNA from four voucher specimens per species from the pool of male *P*. *marginata* and *P*. *dolus* that were originally sequenced and employed to design the SA-HRMA assay. Initial PCR reactions to identify a diagnostic HRMA were performed in 10 µL volumes in glass capillary tubes containing 1x Econotaq Plus Master Mix (Lucigen), 1x LCGreen (Idaho Technologies), 0.2 µM of each primer, and approximately 10 ng of template DNA. Each reaction was covered with ~10 µL of mineral oil to prevent evaporation and ensure uniform melting^13^. A negative control was included in all reactions. Thermocycling was conducted in a RapidCycler2 (Idaho Technologies) with an initial denaturing step of 95 °C for 5 minutes, followed by 45 cycles of denaturing at 94 °C for 10 seconds, annealing at 48 °C for 10 seconds, and extension at 72 °C for 12 seconds, with a final step of melting at 94 °C for 10 seconds and followed by cooling at 40 °C and holding for 20 seconds prior to initiating melting. Products were melted using HR-1 High Resolution Melter (Idaho Technologies), and data was acquired from 65–85 °C with a melting ramp rate of 0.2 °C/s.

**Figure 5:**
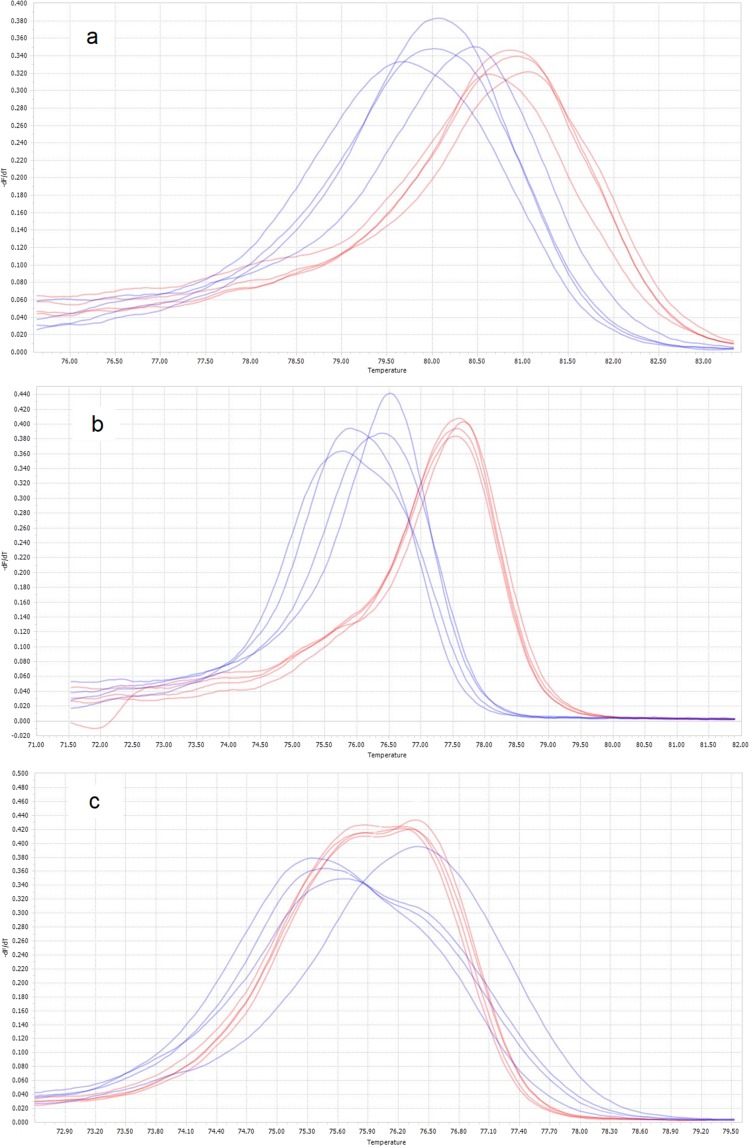
Preliminary HRMA results for three primer sets. The graphs show normalized derivative plots of fluorescence with respect to temperature (−dF/dT). In all panels, *P*. *dolus* is shown in blue while *P*. *marginata* is shown in red. Primer sets shown are (**a**) Prok-HRMA1, (**b**) Prok-HRMA2, and (**c**) Prok-HRMA3. Results for primer set Prok-HRMA4 are given in Fig. 1.

HRMA were carried out with the four primer sets. Five additional HRMAs were tested by combining forward and reverse primers from the original four sets (not shown). The primer pair Prok-HRMA4-F (5′ CCA GTA CTT GCA GTT GCA 3′) and Prok-HRMA4-R (5′ GTT GAT ATA AGA TTG GAT CTC C 3′) was identified as the most successful set of primers to use in HRMA, based on highest amplification efficiency and greatest differences in melting temperatures between species (Fig. 1) compared to the other three sets (Fig. 5). This HRMA was then used to diagnose a larger sample (n = 518) consisting of 279 males that based upon morphological traits were putatively identified as either *P*. *marginata* (n = 164) or *P*. *dolus* (n = 115), and of 239 unidentified females and juveniles of these two species (see Fig. 3).

PCR amplifications for high throughput HRMA were performed in 10 µL volumes containing 1x Econotaq Plus Master Mix (Lucigen), 1x LCGreen (Idaho Technologies), 0.2 µM of each primer, and approximately 10 ng of template DNA. Negative controls were included in all reactions. Thermocycling, performed on a LightCycler 96 Real-Time PCR system (Roche Diagnostics), consisted of an initial denaturing step at 95 °C for 2 min; followed by 55 cycles of denaturing at 95 °C for 10 sec, annealing at 47 °C for 10 sec, and extension at 72 °C for 30 sec. Prior to melting curve analysis data acquisition, products were denatured at 95 °C for 30 seconds, followed by rapid cooling (ramp rate = 2.2 °C/sec) to 40 °C and held there for 30 seconds. Products were then melted, and data was acquired at a rate of 20 acquisitions/°C from 65–85 °C with a melting ramp rate of 0.05 °C/s.

Finally, to determine whether species identification could be carried on different RT-PCR platforms, we conducted this SA-HRMA assay successfully on Idaho Technology’s Rapidcycler-2 and HR-1 machine, Roche’s Lightcycler 460 and Lightcycler 96, and in a Phoenix MyGo Mini, all using the same chemistry and the same, or similar thermocycling and melting profiles (data available upon request).

## Supplementary information

Supplementary Figures

Supplementry Information

## Data Availability

The datasets generated during the current study are available from the corresponding author on reasonable request.
