# Supplementary material for: Genetic species identification of ecologically important planthoppers (Prokelisia spp.) of coastal Spartina saltmarshes using High Resolution Melting Analysis (HRMA)
Source: Sci Rep. 2019 Dec 27;9:20073. doi: 10.1038/s41598-019-56518-4 (PMC6934748; doi:10.1038/s41598-019-56518-4)
Supplement: Supplementary file 1 — Supplementary Figures [file 41598_2019_56518_MOESM1_ESM.pdf]

Genetic species identification of ecologically important planthoppers (*Prokelisia* spp.) of coastal *Spartina* saltmarshes using High Resolution Melting Analysis (HRMA)

G. Janelle Espinoza*[1]

Jaime R. Alvarado Bremer[1, 2]

[1]Texas A&M University at Galveston, Department of Marine Biology, 1001 Texas Clipper Road, Galveston, TX 77554-2888, USA

[2]Texas A&M University, Department of Wildlife and Fisheries Sciences, 210 Nagle Hall, Texas A&M University, College Station, TX 77843-2258, U.S.A.

Corresponding author: G. Janelle Espinoza (Email: Janelle.Espinoza0@gmail.com; Phone: 409.741-4357; Fax: 409.740.5001)

Supplementary Figure S1. Magnified view of the styles of male specimens of (a) *P. marginata* and (b) *P. dolus*. The difference in shape, i.e. curved in *P. marginata* versus angular in *P. dolus*, was the main diagnostic feature used to identify male individuals to species level for this study. These images were taken with a Nikon AZ100M microscope with motorized body and compiled via Nikon BR software.

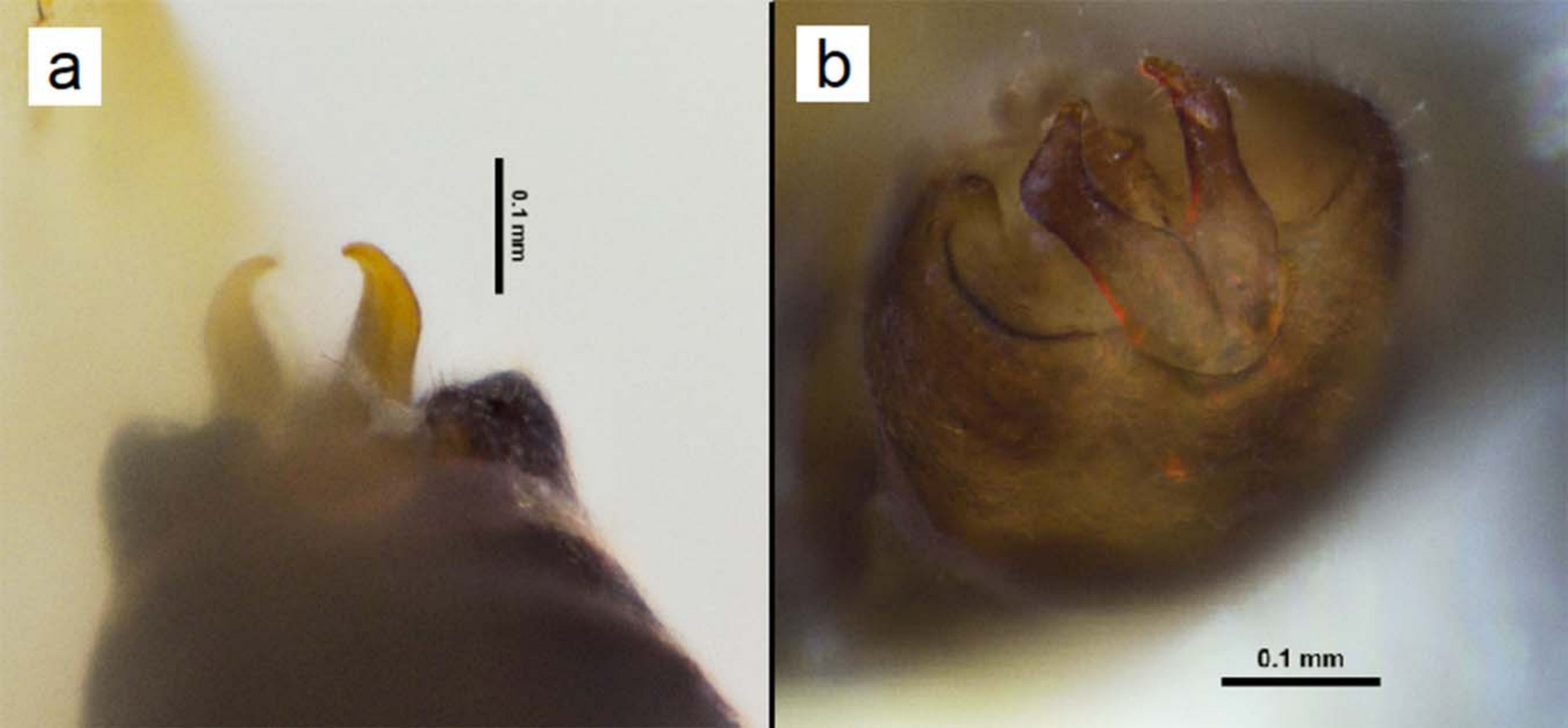
a
0.1 mm
b
0.1 mm
