# Supplementary material for: Genetic species identification of ecologically important planthoppers (Prokelisia spp.) of coastal Spartina saltmarshes using High Resolution Melting Analysis (HRMA)
Source: Sci Rep. 2019 Dec 27;9:20073. doi: 10.1038/s41598-019-56518-4 (PMC6934748; doi:10.1038/s41598-019-56518-4)
Supplement: Supplementary file 2 — Supplementry Information [file 41598_2019_56518_MOESM2_ESM.pdf]

Genetic species identification of ecologically important planthoppers (*Prokelisia* spp.) of coastal *Spartina* saltmarshes using High Resolution Melting Analysis (HRMA)

G. Janelle Espinoza*[1]

Jaime R. Alvarado Bremer[1, 2]

[1]Texas A&M University at Galveston, Department of Marine Biology, 1001 Texas Clipper Road, Galveston, TX 77554-2888, USA

[2]Texas A&M University, Department of Wildlife and Fisheries Sciences, 210 Nagle Hall, Texas A&M University, College Station, TX 77843-2258, U.S.A.

Corresponding author: G. Janelle Espinoza (Email: Janelle.Espinoza0@gmail.com; Phone: 409.741-4357; Fax: 409.740.5001)

Supplementary Figure S2. Consensus sequence from multiple sequence alignment of COI segment for 65 morphologically validated male specimens of *P. marginata* (n=43) and *P. dolus* (n=22). Forward and reverse primer locations for the four primer sets designed in this study are annotated on the sequence. Ambiguities in the consensus sequence correspond to polymorphic sites within and between the species, and are highlighted in pink and coded in standard IUPAC notation.

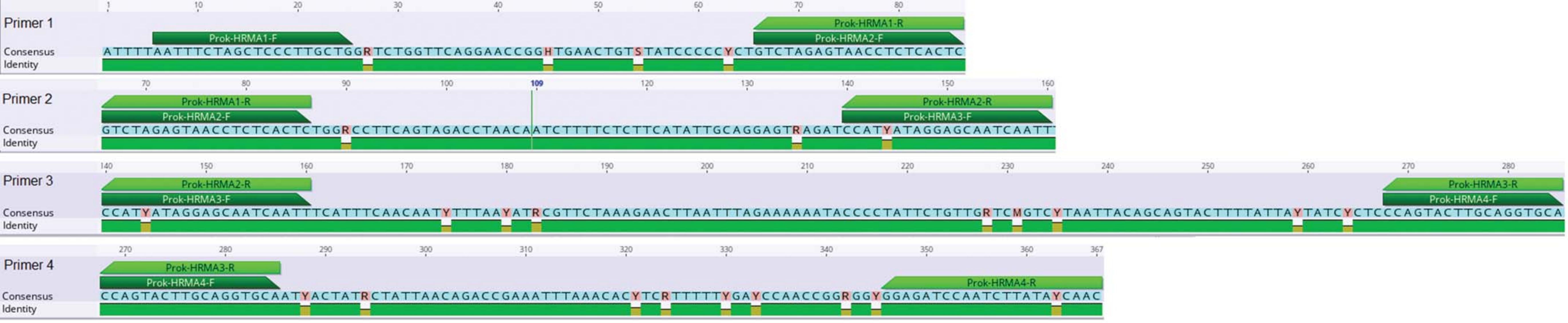

Primer 1
Prok-HRMA1-F
Prok-HRMA1-R
Prok-HRMA2-F
Consensus
ATTTTAATTTCTAGCTCCCTTGCTGGRTCTGGTTCAGGAACCGGHTGAACTGTSTATCCCCCYCTGTCTAGAGTAACCTCTCACTC
Identity
Primer 2
Prok-HRMA1-R
Prok-HRMA2-F
Prok-HRMA2-R
Prok-HRMA3-F
Consensus
GTCTAGAGTAACCTCTCACTCTGGRCCTTCAGTAGACCTAACAATCTTTTCTCTTCATATTGCAGGAGTRAGATCCATYATAGGAGCAATCAATT
Identity
Primer 3
Prok-HRMA2-R
Prok-HRMA3-F
Prok-HRMA3-R
Prok-HRMA4-F
Consensus
CCATYATAGGAGCAATCAATTTCATTTCAACAATYTTTAAYATRCGTTCTAAAGAACTTAATTTAGAAAAAATACCCCTATTCTGTTGRTCMGTCYTAATTACAGCAGTACTTTTATTAYTATCYCTCCCAGTACTTGCAGGTGCA
Identity
Primer 4
Prok-HRMA3-R
Prok-HRMA4-F
Prok-HRMA4-R
Consensus
CCAGTACTTGCAGGTGCAATYACTATRCTATTAACAGACCGAAATTTAAACACYTCRTTTTTYGAYCCAACCGGRGGYGGAGATCCAATCTTATAYCAAC
Identity
